# A novel SYNE1 gene mutation in a Chinese family of Emery-Dreifuss muscular dystrophy-like

**DOI:** 10.1186/s12881-017-0424-5

**Published:** 2017-06-05

**Authors:** Zuzhi Chen, Zhixia Ren, Wenli Mei, Qiankun Ma, Yingying Shi, Yuanxing Zhang, Shujian Li, Li Xiang, Jiewen Zhang

**Affiliations:** Department of Neurology, People’s Hospital of Zhengzhou University, No. 7 Weiwu Road, Zhengzhou, Henan 450003 China

**Keywords:** Emery-Dreifuss muscular dystrophy, *SYNE1* gene, Gly2304Arg mutation, Clinical manifestations

## Abstract

**Background:**

In the present study, a novel mutation in exon 46 at codon 2304 (G2304R) of the *SYNE1* gene is described in a Chinese family (proband, mother, and sister) with Emery–Dreifuss muscular dystrophy-like, which clinically manifests as muscle weakness, muscle atrophy, joint contracture, and without significant cardiac abnormalities.

**Methods:**

Clinical examination and neuroimaging of the captured target region and high-throughput sequencing were performed in a family of four generations. Muscle changes were evaluated using magnetic resonance imaging and muscle biopsies.

**Results:**

Target region capture sequencing yielded a novel missense mutation in codon 2304 (G2304R), which is a heterozygous A to G point mutation at position 6910 (c.6910A > G) in exon 46 of *SYNE1* leading to a glycine-to-arginine substitution (p.Gly2304Arg). The results were also identified by Sanger sequencing in three family members but not in the other three unaffected family members and 100 control subjects.

**Conclusions:**

This mutation is probably pathogenic and is the first of its kind reported in a familial Emery–Dreifuss muscular dystrophy-like.

## Background

Emery–Dreifuss muscular dystrophy (EDMD), characterized by joint contracture, muscle weakness, and cardiac abnormality, is a genetically heterogeneous disease occurring in X-linked and autosomal dominant forms, with various gene mutations. Currently, EDMD is associated with at least seven gene mutations, of which *SYNE1* mutation is relatively less common [1]. The *SYNE1* gene has an autosomal dominant inheritance pattern, and its mutations might result in defects in the expression product nesprin-1. This protein is ubiquitously expressed in multiple tissues and tethers the outer membrane of the nuclear envelope (NE) to the cytoskeleton via interaction with F-actin [2], but particularly abundant in the striated muscles and cerebellum [3]. Therefore, *SYNE1* gene mutations could cause EDMD4 [4] and spinocerebellar ataxia type 8 [5] theoretically, as well as myogenic arthrogryposis multiplex congenita with EDMD features [6] and intellectual disability with spastic paraplegia and axonal neuropathy [7]. In this study, we report a novel *SYNE1* mutation in codon 2304 in a dominant pattern of muscular dystrophy with muscle weakness without significant cardiac abnormalities (“EDMD-like” phenotype). To the best of our knowledge, this mutation (Gly2304Arg), which causes a glycine to arginine substitution, has never been reported previously.

## Methods

### Patients

**Fig. 1:**
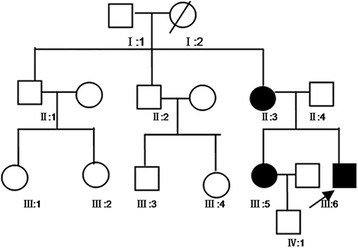
Pedigree of the familial Emery–Dreifuss muscular dystrophy with SYNE1 Gly2304Arg mutation. The *arrow* indicates the proband; *circle*, female; *square*, male; *Slashed* symbol, deceased family member; *black symbol*, affected family member; Blood samples were available from the proband (III:6) and family members (II:3, II:4, III:3, III:4 and III:5)

A total of six individuals from a family of four generations were assessed (Fig. 1), alongside 100 unrelated normal controls matched for age and gender. All participants provided informed consent before enrolment. The experimental protocol was approved by the Institutional Review Board of People’s Hospital of Zhengzhou University.

### Target region capture and analysis

**Fig. 2:**
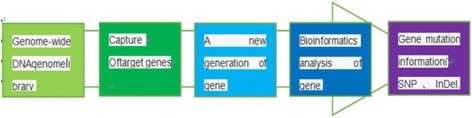
Process of target region capture the high-throughput sequencing. Screening for mutations in the target genes was performed by next generation sequencing (NGS) (Illumina HiSeq 2500, USA) with ≥ 200-fold average high-throughput sequencing depth; mutations were analyzed by bioinformatics

**Fig. 3:**
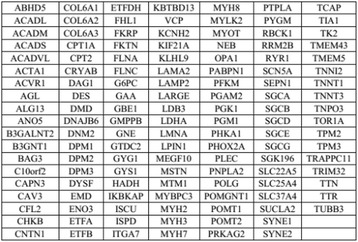
Analysis of the gene list

5 mL blood samples were collected from each subject for genomic DNA extraction using QIAamp DNA Blood Mini Kit (Qiagen, Hilden, Germany), according to the manufacturer’s instructions, and stored at −20 °C. Target region capture and high-throughput sequencing were examined in the proband (III:6) for 131 previously reported genes associated with neuromuscular disease (Figs. 2 and 3). Briefly, point mutations and insertions/deletions were designed for target genes encompassing all exons and splicing sites immediately adjacent to intron sequences. Screening for mutations in the target genes was performed by next generation sequencing (NGS) (Illumina HiSeq 2500, USA) with ≥ 200-fold average high-throughput sequencing depth; mutations were analyzed by bioinformatics.

### Sanger sequencing

Sanger sequencing was used to detect DNA sequence variants; then, the target site primer was designed based on the location of the *SYNE1* gene variant. 50 μL aliquots of standard polymerase chain reaction (PCR) reactions contained 25 μL Premix Taq, 2 μL each forward and reverse primers, 5 μL genomic DNA as template, and 16 μL ddH_2_O. To assess PCR efficiency, 8 μL of amplified products were analyzed by 2% agarose gel electrophoresis. PCR primers were: forward 5′-GGAGCTGGCTTCCATGATGAG-3′ and reverse 5′-ACGTGTTGCTTACTTTGCTGT-3′. PCR amplification was performed on MultiGene OptiMax (Labnet, USA) at 94 °C for 30 s, 54 °C for 30 s, and 72 °C for 1 min. Subsequently, the PCR products of the proband and five family members were purified and directly sequenced on an automated sequencer. Sequencing reads were compared with the Chromas software and NCBI BLAST.

## Results

### Case description

**Fig. 4:**
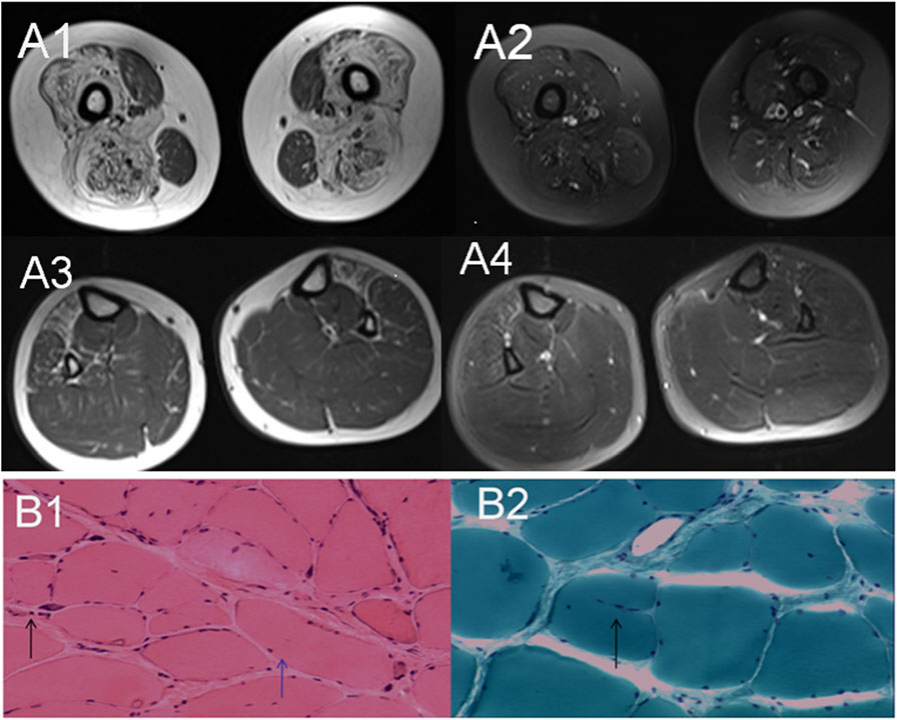
A1-4: The MRI of proband. A1-2 MRI revealed the bilateral thigh muscle disappear, the normal form was filled with a wide range of adipose tissue; A3-4 MRI showed the anteromedial calf group of muscles was infiltrated mild fatty. B1-2: Physical performance of proband; B1 (HE, ×100): the pathological changes in muscles showed that the sizes of muscle fibers were different, fiber atrophy (*black arrow*) and hypertrophy (*blue arrow*) alternately existed, compensatory hypertrophy of partial muscle fibers existed, fat and connective tissue proliferations were apparent. B2 (MGT, ×200): split muscle fibers can be seen in the hypertrophy muscle fibers (*black arrow*)

**Fig. 5:**
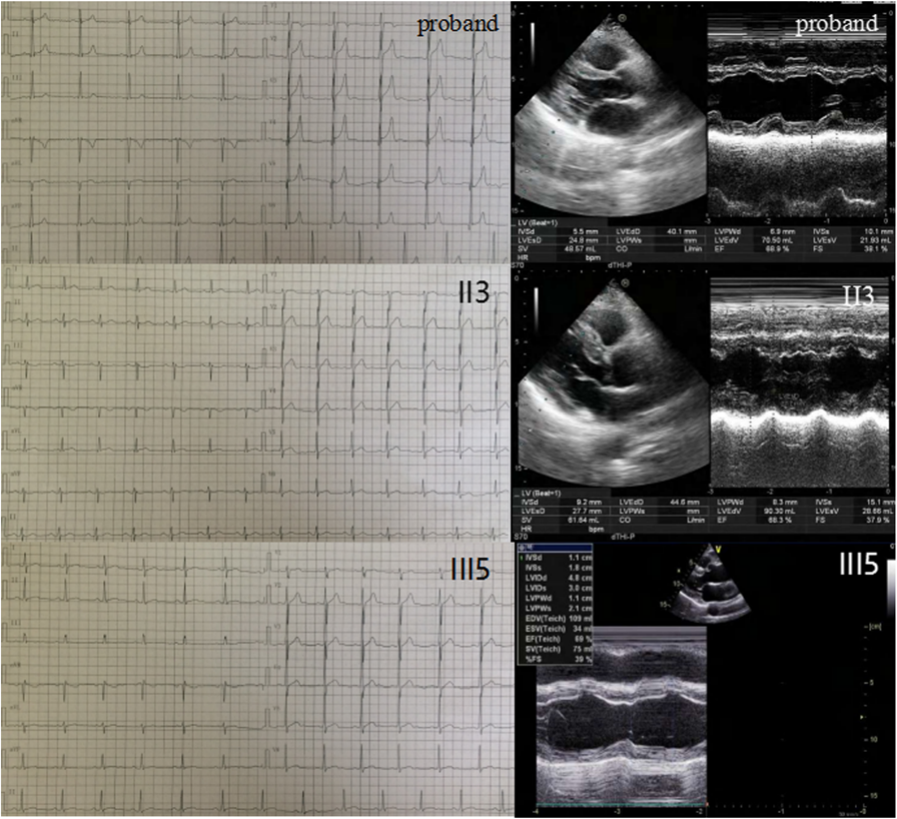
The ECG and Echo of proband、II:3and III:5. Clinical cardiac evaluation showed no abnormalities

The proband (III: 6) was a 24-year-old male. From 12 years of age, he had progressive and bilateral weakness of proximal lower limbs, and proximal muscles of the extremities gradually degenerated. Gradually, he experienced fatigue after walking a short distance, which was slowly accompanied by waddling gait. Subsequently, the neck could not move forward, and the elbow could not be kept straight. Contractures of the bilateral ankle, knees, and elbows appeared. Then, he started to pull himself up by gripping handrails while ascending the stairs, at the age of 16 years. He had difficulties standing up after squatting on the ground. The symptoms mentioned above worsened progressively. At neurological examination, the patient had normal intelligence. The neck could not move forward, and head activities were limited. The bilateral ankle, knee and elbow showed contracture. The hip, wrist, and shoulder were roughly normal. The biceps brachii and quadriceps femoris had significantly shrunken. Muscle tension of both upper limbs was normal, and that of both lower limbs was reduced. The proximal muscle strength of both upper limbs was grade 4; the distal muscle strength was grade 5. The bilateral biceps, triceps, and tendon reflexes had disappeared, and radial periosteal reflex on both sides could be derived. Bilateral knee and ankle reflexes had also disappeared. Relevant laboratory results showed serum muscular enzyme levels distinctly increased (3252.0 u/L), elevated by 17-fold. Blood lactic acid levels were increased slightly (3.5 mmol/L). Magnetic resonance imaging examination of muscles showed bilateral thigh muscle disappearance; muscles were filled with large amounts of adipose tissue while the anteromedial calf group of muscles were infiltrated mildly with fat (Fig. 4 A1-4). Electromyography revealed normal nerve conduction and typical muscle-derived changes, such as low amplitude, broad phase, and significantly increased polyphasic wave. Electron microscopy demonstrated that the size of muscle fibers (obtained by biceps brachii biopsy) was not uniform, and fiber atrophy and hypertrophy existed alternately. Compensatory hypertrophy of partial muscle fibers was found, with apparent fat and connective tissue proliferations. Split muscle fibers could be seen in the hypertrophy muscle fibers (Fig. 4 B1-2). Cardiac examinations were normal (Fig. 5). Patient 2 (III: 5) was a 28-year-old female with proximal weakness of bilateral lower limbs. Neurological examination showed muscle wasting. The clinical cardiac evaluation revealed no abnormalities (Fig. 5). Patient 3 (II: 3) was a 53-year-old female with proximal weakness of bilateral lower limbs, and would feel fatigued after slowly walking a short distance. Clinical cardiac evaluation showed no abnormalities (Fig. 5).

### Mutation analysis

**Fig. 6:**
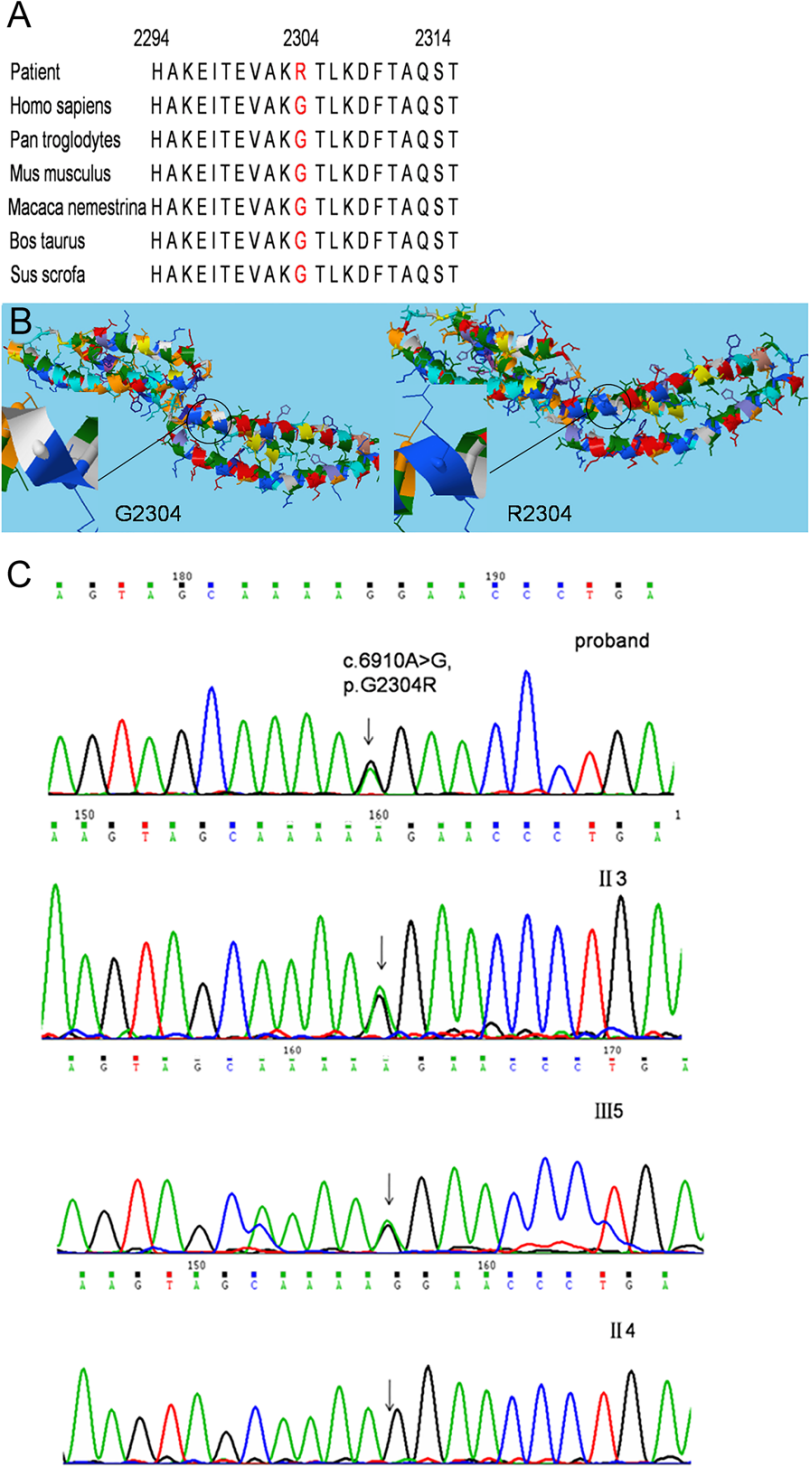
**a** Evolutionary conservation analysis. Protein sequences of Homo sapiens (NP_006715473), Pan troglodytes (NP_016812220), Mus musculus (NP_011241489), Macaca nemestrina (NP_011751944), Bos Taurus (NP_010806995), Sus scrofa (NP_013847930) were retrieved from protein BLAST. **b** Secondary structures of SYNE1 with native Gly 2304 residue and Arg mutation. The structures of nesprin-1 with native Gly 2304 residue and Arg mutation were generated by Raptor 3D prediction program. The wild-type and mutant residues were colored with *white* and *blue*. Mutation p.G2304R altered the side chain of residue at position 2304. (For interpretation of the references to color in this figure legend, the reader is referred to the Web version of this article). **c** DNA sequencing chromatograph of exon 46 of the SYNE1 gene (abnormal and normal), SYNE1 gene with a heterozygous mutation: c. 6910 A > G (p. G2304R). The arrow pointed the mutation

Target region capture sequencing showed a heterozygous missense mutation in exon 46 of the *SYNE1* gene (NM_033071, c.6910A > G, p.G2304R) of the proband, which lead to a glycine-to-arginine substitution (p.Gly2304Arg) that has been associated with the EDMD-like phenotype. The mutation were present in two other members of the family (II3 and III5) (Fig. 6c) but not in the other 3 family members or the 100 Chinese individuals as healthy controls. The mutation was not detected in the remaining 3 pedigrees or the 100 healthy individuals. This missense mutation was not reported in The Human Genetics Mutation Database (http://www.hgmd.org; [8]) and previous literatures. In addition, the mutation was not a single nucleotide polymorphism. Furthermore, mutation analysis by the Polyphen-2 online software and MutationTaster, HumDiv (sensitivity: 0.86; specificity: 0.91) and HumVar (sensitivity: 0.88; specificity: 0.75) predicted potentially damaging effects, which indicated that the missense mutation might be pathogenic. The SYNE1 protein sequences of six mammalian species, including human, were examined and analyzed by NCBI GenBank (Fig. 6a). The glycine at position 2304 of the SYNE1 protein was located in a highly conserved region. The more mutations in the conserved region, the more pathogenic the gene. Therefore, we hypothesized that G2304R may be a pathogenic mutation. This mutation altered the side chain of the 2304 amino acid residue as assessed by the Raptor 3D online software (Fig. 6b). These results demonstrated that the novel mutation was the most probable cause of the familial EDMD-like.

## Discussion

**Fig. 7:**
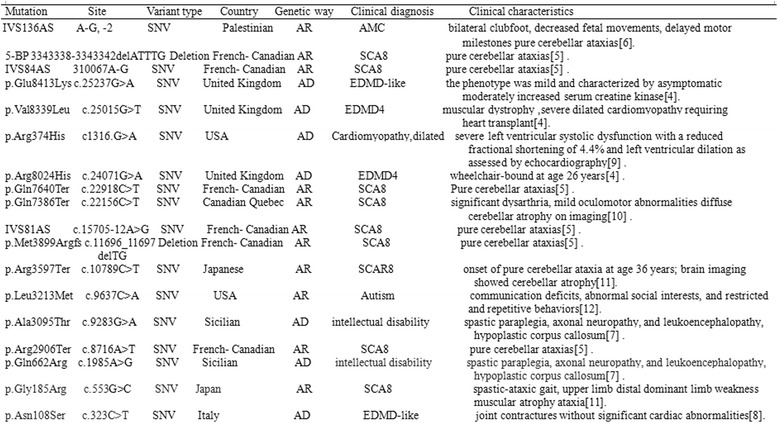
Clinical features of reported SYNE1 mutations in the all word. SNV: single nucleotide variant; AMC : Arthrogryposis multiplex congenita ; EDMD4: Emery-Dreifuss muscular dystrophy 4; SCA8: Spinocerebellar ataxia8; [4]: References [4–7, 9–13]

We reported a family including three patients (the proband and his elder sister and mother) affected with progressive muscular dystrophy manifested by joint contracture without cardiac involvement (“EDMD-like” phenotype). Herein, we identified a novel missense mutation in exon 46 of the *SYNE1* gene. The pathologic nature of the Gly2304Arg mutation was supported by the phylogenetic conservation of the residue. Multiple sequence alignment for *SYNE1* was performed in comparison with different organisms, and evolutionary conservation analysis indicated that the novel p.G2304R mutation resulted in a highly conserved amino acid change. These analyses revealed its predicted deleterious effects, localization in a functionally crucial domain, absence in multiple control chromosomes, and no other mutations found by NGS in the family. On the other hand, pathogenicity prediction for G2304R showed probable damaging effects, as assessed by Polyphen-2. Thus, a series of concordant clues suggested a primary role for the *SYNE1* mutation identified in the pedigree. Presently, fewer *SYNE1* gene mutations are known in comparison with other gene mutations in EDMD patients. In the Human Genetics Mutation Database (HGMD) and ClinVar Database, mutation sites described in the *SYNE1* gene regarding clinical features are summarized in Fig. 7. Comparing the current cases with previously reported mutations, we found that the pedigree in this study presented the clinical manifestations of the EDMD-like phenotype.

EDMD is typically characterized by the following features: (1) early contractures of the Achilles tendons, elbows, and post-cervical muscles, with subsequent severe contracture causing spinal column anteflexion limitation; (2) progressive skeletal muscle weakness and muscle wasting at disease onset; (3) life threatening cardiac conditions, as the most severe complications. This may be attributed to myocardial cell replacement by adipose and fiber tissues, resulting in abnormal conduction and systolic functions of the myocardium, with ventricular and supraventricular arrhythmias, chamber dilation, and heart failure [5]. In the present study, the clinical manifestations of the proband (III: 6) were muscle weakness, muscle atrophy, and joint contracture. However, his mother (II: 3) and elder sister (III: 5) only presented muscle weakness and muscle atrophy. The three patients had normal heart function as evaluated clinically. Taken together, the pedigree’s clinical features reflected an “EDMD-like” phenotype. Two patients with a similar clinical phenotype but two different *SYNE1* gene mutations have been reported previously [4, 9].

Muscle pathology in EDMD patients shows nonspecific changes such as non-uniform muscle fiber size, increased nuclear muscle, connective tissue, and muscle fiber necrosis. In the current case, pathological alterations were different muscle fiber sizes, fiber atrophy and hypertrophy existing alternately, compensatory hypertrophy of some muscle fibers, and apparent fat and connective tissue proliferation. Split muscle fibers could be seen in hypertrophic muscle fibers. Hence, these changes were consistent with those previously reported [9]. Electrophysiological tests showed typical muscle-derived variations in previous studies for most patients. Consistently, the electrophysiological tests of the current proband showed muscle-derived damage.

## Conclusion

In summary, we reported a novel mutation in exon 46 on codon 2304 (G2304R) of the *SYNE1* gene in a Chinese family (proband, mother, and sister) with EDMD-like features, and 100 healthy individuals did not show such mutation. The current study had a few limitations. First, whether other members of the family were mutation site carriers was not assessed. In addition, the patients and their families do not want to check contractures and neck muscle examination due to financial reasons. Furthermore, analysis of the nuclear proteins emerin and lamin A/C by muscle immunofluorescence was not performed. Finally, further verification of the mutations at the protein level is required.

## Abbreviations

EDMD: Emery-Dreifuss muscular dystrophy
HGMD: Human genetics mutation database
MRI: Magnetic resonance imaging
NE: Nuclear envelope
PCR: Polymerase chain reaction
SNPs: Single nucleotide polymorphisms
